# Animal Models of Diabetic Retinopathy

**DOI:** 10.1007/s11892-017-0913-0

**Published:** 2017-08-24

**Authors:** Ana Maria Olivares, Kristen Althoff, Gloria Fanghua Chen, Siqi Wu, Margaux A. Morrisson, Margaret M. DeAngelis, Neena Haider

**Affiliations:** 1 Schepens Eye Research Institute, Massachusetts Eye and Ear Infirmary, Department of Ophthalmology, Harvard Medical School, 20 Staniford Street, Boston, MA 02114 USA; 2 Moran Eye Center, University of Utah, Salt Lake City, UT 84132 USA

**Keywords:** Animal models, Diabetic retinopathy, Diabetes, Induced models, Pancreatectomy, Alloxan, STZ, Genetic models, VEGF

## Abstract

**Purpose of Review:**

Diabetic retinopathy (DR) is one of the most common complications associated with chronic hyperglycemia seen in patients with diabetes mellitus. While many facets of DR are still not fully understood, animal studies have contributed significantly to understanding the etiology and progression of human DR. This review provides a comprehensive discussion of the induced and genetic DR models in different species and the advantages and disadvantages of each model.

**Recent Findings:**

Rodents are the most commonly used models, though dogs develop the most similar morphological retinal lesions as those seen in humans, and pigs and zebrafish have similar vasculature and retinal structures to humans. Nonhuman primates can also develop diabetes mellitus spontaneously or have focal lesions induced to simulate retinal neovascular disease observed in individuals with DR.

**Summary:**

DR results in vascular changes and dysfunction of the neural, glial, and pancreatic β cells. Currently, no model completely recapitulates the full pathophysiology of neuronal and vascular changes that occur at each stage of diabetic retinopathy; however, each model recapitulates many of the disease phenotypes.

## Introduction

Diabetic retinopathy occurs in approximately one third of people with diabetes [1]. It is the leading cause of blindness in adults aged 24–70 [1, 2]; in 2010, an estimated 92.6 million adults had diabetic retinopathy (DR), of which 28.4 million individuals experience vision impairment associated with DR [3]. The total prevalence of DR appears to be higher in patients with type 1 than in those with type 2 diabetes [3, 4]. Approximately 25% of patients with type 1 diabetes start to develop symptoms of DR within 5 years after diabetes onset, and the number increases to 80% by 15 years [5]. Interestingly, while sex has not been found to impact susceptibility of type 1 or 2 [3], race [6–9] and socioeconomic status [8, 10] do influence susceptibility for DR. In 2006, the Multi-Ethnic Study of Atherosclerosis (MESA) reported disparities in DR prevalence between diabetic patients of different racial backgrounds: 36.7% in African-Americans, 37.4% in Hispanics, 24.8% in Caucasians, and 25.7% in Chinese-Americans [8, 9]. As a complex disease, it is clear that DR is strongly influenced by both genetics and environment [9, 11••, 12–17]. Overall, the number of patients suffering from DR is expected to rise due to increasing prevalence of diabetes and longer life expectancies for patients with diabetes [3].

**Fig. 1:**
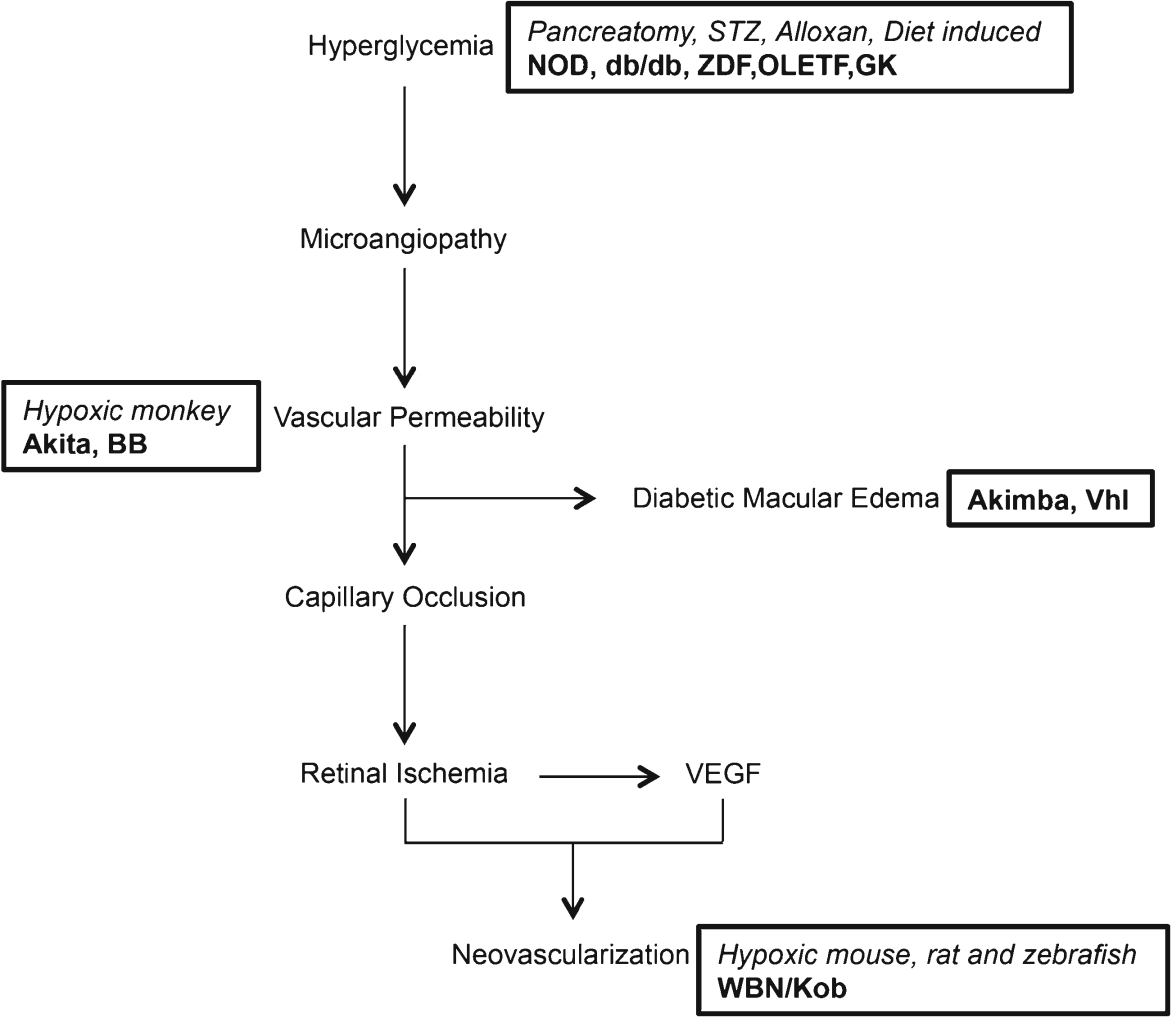
Schematic representation of diabetic retinopathy (DR) disease progression. DR initiates with hyperglycemia, which induces microangiopathy. This leads to vascular permeability, followed by diabetic macular edema and capillary occlusion. Capillary occlusion leads to retinal ischemia, followed by elevated levels of VEGF, ultimately resulting in neovascularization. *Boxes* represent the point in the pathway targeted by animal models. *Italicized text* corresponds to the induced models and *bold text* corresponds to the genetic models

The onset and progression of DR is triggered by numerous factors including extended duration of diabetes, poor control of blood glucose, and elevated blood pressure [9]. Hyperglycemia leads to the development of microangiopathy, including microaneurysms, hemorrhages, and basement membrane thickening [18, 19]. This results in increased vascular permeability of the blood-retinal barrier (BRB) causing leakage and diabetic macular edema (DME) [1, 18, 19]. Vascular permeability also causes increased capillary occlusion that leads to retinal ischemia, triggering an increase in the levels of vascular endothelial growth factor (VEGF) [1, 18]. Retinal ischemia and elevated VEGF levels then promote neovascularization [1, 18]. A schematic summary of various factors that contribute to disease progression is depicted in Fig. 1.

DR is classified as either nonproliferative (NPDR) or proliferative (PDR) based on the presence of neovascularization that typifies the proliferative form [1, 20]. NPDR, which can progress to preproliferative DR, exhibits microaneurysms, dot and blot hemorrhages, cotton-wool spots, and capillary nonperfusion due to microvascular damage and pericyte loss. Microglial changes and DME can also occur in NPDR. In PDR, neovascularization results in retinal and vitreous hemorrhages and can lead to retinal detachment. DME may also occur in PDR [1]. Disease models have contributed greatly to the understanding of mechanisms that lead to DR disease.

Several animal models have been developed to investigate the etiology and pathogenesis of DR and to develop and test therapies to treat the disease. As DR is a complex disease with both genetic and environmental influences, animal models are similarly developed by induction or genetic mutation. Induced models are generated through surgery, drugs, diet, and laser or chemical damage. Genetic models are created using selective breeding and gene editing. While a large selection of species have been used to generate DR models, including mice, rats, cats, dogs, pigs, and nonhuman primates, mouse and rat models are most often studied, as their small size, short life span, and fast breeding rates allow for the most efficient studies. Rodents have also been the focus of most genetic studies, with the discovery of inherited hyperglycemia or obesity particular to certain strains [21, 22, 23•, 24–26]. DR phenotypes in dog models, however, appear to be most similar to human DR [27–30]. Surprisingly, nonhuman primates have proven relatively resistant to induced DR [31]. Cats generally do not develop cataracts [32]. Pigs and zebrafish, in contrast, are preferred for the similarity of their eye structure to humans, easily visualized vascular structures, short life spans, and large breeding sizes (zebrafish) [27, 33, 34]. Though no single animal model to date represents the complete range of vascular and neural complications of human DR in both early and late stages, the models described in this review have been instrumental in determining the mechanisms behind DR in the hopes of developing novel therapies.

### Induced Models

Induced models have been created through five methods: surgical removal of the pancreas, administration of the drug alloxan, administration of the drug streptozotocin (STZ), high-galactose diets, and laser or chemical damage to the eye [27, 35–46]. While all methods of induction are still studied today, the most common is STZ administration, as it results in the fastest rate of disease development [41]. Alloxan is considered to be less efficient in diabetic induction, and dietary methods require the most time for disease progression [43]. Surgery- and damage-induced models are the most technically challenging, limiting their use historically. The most frequently used models for inducing DR are mice and rats, but dogs, cats, pigs, rabbits, monkeys, and zebrafish are also used. Presentation of induced DR pathology is generally slower in larger animals, making rodents and, recently, zebrafish more favored models. A comparison of the available induced models can be found in Table 1.

**Table 1:** Phenotypes of induced and genetic animal models of DR

	Hyperglycemia	Damaged pancreatic beta-cells	Damaged/decreased pericytes	Microglial changes	ONL, INL, or IPL thinning	RGC loss	Endothelial cell loss or damage	Basement membrane thickening	Microaneurysms	Hemorrhages	Increased vascular permeability	BRB leakage or breakdown	Macular edema	Increased acellular capillaries	Neovascularization	Retinal detachment	Comments and references
Induced models																	
Pancreatectomy																	
Dog	X	X								X							[35–37]
Cat	X	X						X	X						X		Capillary nonperfusion [47–49]
Monkey	X	X										X					[50]
Alloxan																	
Mouse	X	X	X	X		X			X					X			Functional defects [51, 52]
Rat	X	X	X	X				X				X		X	X		Endothelial swelling [53–55, 59, 60]
Dog	X	X	X						X	X				X			[28]
Pig	X	X	X	X													Capillary collapse [61]
STZ																	
Mouse	X	X	X	X	X	X								X	X		Increased astrocytes [27, 56, 63 71–73]
Rat	X	X	X			X	X	X			X	X		X			[67, 68, 74, 75]
Zebrafish	X	X			X												[69, 70]
Rabbit	X	X								X							Vascular lesions [27, 64]
Dog*	X	X	X					X									*Alloxan/STZ-induced [68, 76]
Monkey	X	X								X				X			Ischemic retinopathy [27, 31]
Pig	X	X			X	X		X			X						[65, 66, 77]
Diet																	
Mouse	X		X				X	X	X					X			[27, 44, 78, 79]
Rat	X		X	X				X	X					X			[20, 60, 80]
Rabbit	X								X	X							[27, 81]
Dog	X		X					X	X	X				X	X		[43]
Zebrafish	X				X												[82]
Hypoxic damage																	
Mouse									X						X		Nonperfusion [86–88]
Rat					X										X		Abnormal vascular tufts [89–93]
Monkey									X	X	X				X		Nonperfusion [96]
Zebrafish															X		[94, 95]
Genetic models																	
Mouse																	
Ins2^Akita^		X		X	X	X			X	X	X			X			*Insulin2* [99–104]
NOD	X	X	X		X	X		X									Disorder focal proliferation vessels [105–113]
db/db	X	X	X			X		X	X		X	X					*Lepr* [24, 25, 114–120]
Kimba			X		X												*Vegf* [121–124]
Akimba			X		X				X		X		X		X	X	*Insulin2*, *Vegf* [125, 126]
Rat																	
BB		X	X					X	X			X					*Ian4* [135–144]
ZDF	X		X					X									*Lepr*; increased capillary density [128, 129]
OLETF	X		X				X	X	X								*GPR10*; leukocyte entrapment [131–134]
WBN/Kob														X	X		QTL Pdwk1 [20, 145–148]
SDT	X		X		X	X											QTL Gisdt1, 2, 3 [149–155]
GK	X																Increased endothelial cells [156–161]
Zebrafish																	
*Vhl* ^*−/−*^													X		X	X	*Vhl*; increased hyaloids [162]

Models shown to present with each phenotype are marked with an X

*DR* diabetic retinopathy, *ONL* outer nuclear layer, *INL* inner nuclear layer, *IPL* inner plexiform layer, *RGC* retinal ganglion cell

#### Pancreatectomy

One of the oldest methods used to induce diabetes in animal models is pancreatectomy, the removal of the pancreas or removal of β cells from the pancreas. Pancreatectomy was observed as early as 1922 to increase blood sugar levels in dogs [35], and by 1968–1971, a technique of complete pancreatectomy in adult dogs had been developed to induce diabetes [36, 37]. This technically difficult method is usually applied to large animals such as cats and monkeys [27]. In adult cats, hyperglycemia develops within 3 weeks postsurgery; this time can be reduced to within 1 week by combining pancreatectomy with administration of alloxan [47] (described in the “Alloxan” section). Thickening of the basement capillary membrane can occur from 3 months after the onset of hyperglycemia [48]. Other DR symptoms, including microaneurysm, intraretinal hemorrhages, capillary nonperfusion, and neovascularization, may take 5–9 years to develop [49]. Maintenance of this model thus requires an extended period of time.

In monkeys, pancreatectomy at various ages between 6 and 15 years resulted in insulin dependency and hyperglycemia, which was then deliberately uncontrolled [50]. This was observed to lead to BRB leakage within 1 year of hyperglycemia onset. However, 10 years postinduction, monkeys still did not develop proliferative retinopathy [50]. Monkeys thus appear to be surprisingly resilient to induction of DR despite removal of the pancreas, long-term diabetes, and poor control of blood sugar levels. This is a similar phenomenon to humans where only 30% of individuals with diabetes develop DR, suggesting primates may have additional biological mechanisms to re-regulate homeostatic state in the presence of chronic insult. Understanding the regulatory factors that contribute to the physiological differences in species is important for developing appropriate disease models.

#### Alloxan

The first drug found to induce diabetes, alloxan, was discovered by Dunn and McLetchie in 1942 [38]. Alloxan is a derivative of uric acid and directly targets β cells found in the pancreas [39], and was first produced by Wöhler and Liebig through a reaction of uric acid with nitric acid. While conducting rabbit studies focused on kidney disorders, Dunn and McLetchie found that intravenous injection of alloxan resulted in hypoglycemia due to necrosis in the islets of Langerhans in the pancreas. Death of β cells led to the release of insulin stores in these cells, causing the observed hypoglycemia followed by onset of diabetes within 24 h. Dunn and McLetchie also created the diabetic rat model induced by alloxan via intraperitoneal administration. While the diabetic rabbits appeared listless and lost weight, rats that were given alloxan ate voraciously and presented with polydipsia, polyuria, glycosuria, and hyperglycemia, characteristic of diabetes [38].

Alloxan-directed cell death is mediated by inhibition of glucokinase, an enzyme involved in the glucose-insulin regulatory pathway and expressed in the liver and pancreas. The drug can be toxic to liver and kidney cells, but with proper dosing, toxicity can be avoided. The action of alloxan in the pancreas is specific to β cells, with no toxic effect on a α, δ, or pancreatic exocrine cells. The compound is also unstable in water at room and body temperature, making it difficult to administer [38, 40]. In recent times, alloxan has fallen in popularity in favor of STZ, described in the “Streptozotocin” section, due to the latter’s greater ease of use and efficacy.

Alloxan has been used to induce DR in a large variety of animals including mice, rats, dogs, and pigs, as well as the rabbits and rats [28, 51•, 52–58]. All models experience damage to pancreatic β cells. Mice aged 8–10 weeks can be given a single dose of alloxan to induce hyperglycemia leading to diabetes [51•]. It was previously believed that the alloxan-induced diabetic mouse did not develop cellular or vascular lesions, but a recent study found that alloxan does induce pericyte ghosts and loss of retinal ganglion cells (RGCs) within 7 days and microaneurysms with increased acellular capillaries by 21 days in mice from the FOT_FB strain [51•]. Alloxan also induced microglial changes, with thicker cell bodies and shorter dendrites by 3 months of age in the same animals [52].

Induction of DR by alloxan in rats is determined by weight (180–200 g weight) [53, 54]. Within a week of alloxan administration, hyperglycemia and diabetes develop [54, 55]. Neovascularization occurs between 2 and 9 months postinduction [54] and cataracts within a year [59]. Similar to the phenotypes observed for mice, pericyte ghosts, acellular capillaries, and thickened capillary basement membrane are observed by 15 months postinduction [59, 60]. In addition, the alloxan-induced diabetic rat exhibits BRB breakdown [55], expansion of Müller glia, and endothelial swelling [54]. This model is typically studied for up to 22 months [60].

Alloxan induces diabetes in young dogs by once a week administration for 5 weeks. This results in retinopathy remarkably similar to DR in humans however, dogs can take up to 53 to 69 months after onset of alloxan diabetes to develop DR [28]. Following disease onset, alloxan-treated dogs present with hemorrhages, acellular capillaries, pericyte loss, and microaneurysms, making this a viable model of PDR. This phenotype persists for 11 months.

The porcine alloxan-induced DR model, in contrast to the dog models, develops hyperglycemia within 48 h [61]. The molecular phenotype following induction is Müller cell contraction-promoting activity that is detectable as early as 30 days after alloxan administration, and sustains for up to 90 days. Alloxan-induced pigs also develop cataracts by 60 days following alloxan administration [61] as well as BRB breakdown, capillary collapse, and pericyte ghosts were detected by 20 weeks [56]. In contrast to other alloxan models that exhibit PDR like DR disease, the porcine alloxan-induced model of DR recapitulates several important markers of NPDR.

#### Streptozotocin

In 1963, Rakieten et al. reported that STZ administration causes diabetes in rats and dogs [41]. STZ is an antibiotic produced by *Streptomyces achromogenes* and was studied for use in cancer chemotherapy [62]. Rakieten et al. studied intraperitoneal administration of STZ in rats and intravenous injection of STZ in dogs, both of which led to sustained hyperglycemia in each species, along with polyuria and polydipsia characteristic of diabetes [41]. The mechanism of diabetes mellitus induction was found to be the disruption of pancreatic islets of Langerhans and loss of β cells due to STZ [41]. β Cells take up STZ specifically because they express the low affinity glucose transporter 2 (GLUT2), and STZ is structurally similar to glucose and *N*-acetyl glucosamine [42]. Other cells that also express GLUT2, including hepatocytes and renal tubular cells, experience similar damage with STZ administration [42]. STZ mechanism of action is cell death by DNA fragmentation.

Induction of DR by STZ has been observed in multiple models including mice, rabbits, pigs, rats, dogs, zebrafish, and monkeys [31, 63-70]. STZ is now generally preferred over alloxan, as it is more effective in recapitulating the diabetic disease state, though both drugs are still commonly used [41]. Several protocols for STZ induction of diabetes in mice have been developed, ranging from 1 to 5 doses delivering a total of 150 to 400 mg/kg of STZ [27]. Hyperglycemia onset typically occurs within 2 weeks, regardless of dosage [27] and can be maintained for up to 22 months [71]. DR phenotypes observed in STZ mice include increased number of astrocytes and gliosis 4–5 weeks after onset hyperglycemia [63, 71], RGC loss at 6 weeks [56], retinal inner nuclear layer (INL) and outer nuclear layer (ONL) thinning at 10 weeks [72], neovascularization at 16 weeks [73], and acellular capillaries and pericyte ghosts at 6 months [71].

In contrast to mice, rats require lower doses of STZ to develop diabetes [27]. The onset of retinal lesions differs between rat strains, but several observed phenotypes include BRB breakdown 2 weeks after diabetes onset [74, 75], ONL thinning beginning the in the fourth week following induction [74], increased acellular capillaries, decreased numbers of both pericytes and endothelial cells after 8 weeks [67], and basement membrane thickening after 1 year [68]. STZ-induced DR rat models are typically studied for up to 20 weeks [75].

While rodents are commonly used for STZ-induced diabetes, several other models have been studied with various outcomes and onset of disease. Adult zebrafish, 4–6 months of age, injected with multiple doses of STZ intraperitoneally or through direct caudal fin injection over one or several weeks, develop hyperglycemia and within 3 weeks, and display inner plexiform layer (IPL) thinning, photoreceptor segment layer (PSL) thinning, cone receptor dysfunction, and neuronal damage by 4 weeks [69, 70]. This model is maintained approximately 80 days after induction of diabetes [70].

Larger animal models such as rabbits, dogs, and nonhuman primates use a single dose protocol for STZ induction. A single dose of STZ can be given to rabbits weighing 1.5 kg to induce hyperglycemia, which after 135 days results in retinal and preretinal hemorrhages, vascular lesions, venous thrombosis, and proliferative retinopathy [27, 64]. Beagles ranging in age from 4.5 to 17 months and weighing between 11 and 24 kg given a single dose alloxan/STZ cocktail develop hyperglycemia within 2 days [68]. Alloxan/STZ-induced diabetic dogs present with basement membrane thickening after 1 year and pericyte ghosts and smooth muscle cell loss after 4–5 years [76]. This model is studied for 7 years [76]. Interestingly, monkeys treated with a single dose of STZ at age 12 develop diabetes, then ischemic retinopathy with cotton-wool spots and hyperfluorescent spots after 10 years [27, 31]. Interestingly, the induction of hypertension is required for retinopathogenesis in this model, as monkeys without hypertension fail to develop retinopathy [31]. The porcine model of STZ is induced, at 20 kg, with STZ administration for three consecutive days [65]. Induced pigs develop hyperglycemia within 1 week and are studied for up to 32 weeks. Diabetes lasting 4–8 months after STZ induction results in increased BRB permeability, thinning of the INL and ganglion cell layer (GCL), and thickening of the capillary basement membrane [65, 77]. When STZ-induced pigs are subject to hyperlipidemic diets, they acquire dyslipidemia similar to that experienced by patients with type 2 diabetes. Diabetic pigs also experience increased BRB permeability, as well as compromised tight junctions in the retina. The pig’s large size and hierarchal vascular structures make its metabolic and circulatory functions highly similar to humans [66], thus making it a common model for DR.

#### High-Sugar Diets

Kern and Engerman first reported an animal model of DR induced by galactose-heavy diet [43]. Several high-sugar diet models have been developed including mice, rats, rabbits, dogs, and zebrafish [33, 43, 44, 60, 78–83] that were persistently exposed to galactose developing retinopathy similar to that observed in human diabetes. Maintenance of galactose exposure results in continued disease progression. However, galactose-fed animals lack some metabolic abnormalities experienced in diabetes [20]. Mice developed hyperglycemia by 6 weeks of age following high-galactose diet [44]. After 15 months of hyperglycemia, endothelial cell loss and increased acellular capillaries were observed [44, 78]. After 21 months, lesions including pericyte ghosts, microaneurysms, and retinal thickening are observed [27, 44, 79]. While retinopathy takes longer to develop in these mice, they do live longer than other models, allowing them to be observed over a longer period of time, up to 26 months [78]. Similarly, rats have been kept on high-galactose diets for over 2 years. Phenotypes observed in rodents on a continuous high-sugar diet include pericyte ghosts, acellular capillaries, and capillary basement membrane thickening by 18 months of hyperglycemia [60, 80], as well as gliosis and microaneurysm by 28 months [20]. While rodents can develop diet-induced DR, drug-induced and genetic models are more commonly studied in small animals due to their faster onset of disease.

In contrast, larger animals generally take longer to develop DR whether by drug induction or diet. Rabbits fed a high-sucrose diet for 24 weeks develop hyperfluorescent dots and microaneurysms appeared by the 12th week of the diet [27, 81]. Dogs fed a diet with 30% increased galactose develop a more complex disease phenotype including DR and cataracts within 1 year; pericyte ghosts, microaneurysms, dot and blot hemorrhages, and acellular capillaries by 32 months; and basement membrane thickening by 60 months [43]. As observed with all dog models of DR, disease can take many years to develop, but phenotypes in the dog are most similar to those in humans [27, 29, 30, 80, 84].

Most recently, hyperglycemic zebrafish have been developed as a model for DR. Zebrafish are housed in freshwater with alternating concentration 0 and 2% glucose every other day and develop hyperglycemia after 28 days and IPL thinning [82]. As this model has only been maintained for 28 days to date, several attributes including similar retinal topography, ease of vascular structures visualization with fluorescent expression [33, 83], short life span, and large breeding size reduce experimental time and make zebrafish a strong model to study DR [27].

#### Hypoxic Damage-Induced Retinopathy

Models of retinal neovascularization and vasculature leakage lacking hyperglycemia have been used in recent years to study DR. These models simulate advanced-stage PDR observed in human patients. In a 1969 study, Dollery, Bulpitt, and Kohner exposed newborn kittens to hyperoxic conditions and found that returning the kittens to normal air made them experience hypoxia, leading to neovascularization [85]. It was later discovered that retinal damage induced the release of angiogenesis factors [45]. This discovery led to a number of different damage models for retinal neovascularization using mouse, rats, primates, and zebrafish [27, 46]. Hyperoxic mouse models are generated by exposing juvenile mice, typically postnatal days 7–12, to hyperoxic conditions, which results in hypoxic conditions of the retina once they return to normal air and the growth of blood vessels in the retina [86, 87]. These models of oxygen-induced retinopathy (OIR) exhibit neovascularization and nonperfusion, accompanied by the appearance of microaneurysms, which typically occur within 5 days postexposure [88].

Similar to mice, OIR in nondiabetic rats results in neovascularization. Rat pups were exposed to the hyperoxic conditions between 11 and 14 days [89, 90]. Neovascularization is apparent immediately once rats are returned to normoxic conditions followed by astrocyte degeneration [91] and subsequent reduction in INL and IPL thickness, with disorganized outer segments [92, 93]. A distinct feature of this model is the incomplete development of the vascular plexus and the presence of abnormal endothelial tufts [91]. Two nonrodent OIR models, monkey and zebrafish, also develop neovascular disease. OIR-induced neovascularization in zebrafish requires for the animal to be placed in normoxic water followed by the gradual reduction of O_2_ tension over a period of 48–72 h until reaching 10% of air saturation (820 ppb) [94]. Zebrafish can be maintained in this environment for up to 15 days [95]. After exposure, neovascularization is evident as well as reduction in intercapillary distance [94]. The primate model of OIR-induced neovascularization is distinct in that induction is localized by laser vein occlusion. Thus, focal regions of hypoxia are created rather than a whole organism exposure to hypoxic conditions. Retinal neovascularization in OIR primates typically occurs 4–7 days postexposure. Hypoxic monkeys show vascular leakage, venous occlusion, capillary nonperfusion, venous dilation, and dot and blot hemorrhages, which result from microaneurysm ruptures [96]. Interestingly, a primate model was used to develop anti-VEGF treatment [96].

#### Cytokine Induction

The alkali burn model results in increased cytokine activity to produce DR like neovascularization. This model is less commonly used and involves a more painful method to induce retinal neovascularization in mice. This technique has been used in inbred mouse strains such as BALB/c and involves placing 2-mm filter disks soaked in 1 N NaOH on the ocular surface adult mice [97]. Neovascularization is typically observed within 2 weeks. Not surprisingly, treated mice also exhibit increased levels of inflammatory cytokines in neovascularized retinas [96]. While cytokines may be secondary to the neovascular disease phenotype, they likely play a significant role in maintaining the disease state.

### Genetic Models

There are several genetic modes of DR in mouse, rat, and zebrafish. These models include spontaneous, strain-specific, and genetically edited mutations. Several inbred mouse strains for example, including the nonobese diabetic (NOD) and db/db (Lepr^db^), exhibit hyperglycemia, one of the main characteristics of diabetes, and are thus maintained and studied as diabetic models. Rodents are frequently used as genetic models of DR as they are easy to maintain, have well-characterized genetic backgrounds, and are easily manipulated to generate knockout or transgenic models. Genetic models exist for both type 1 and type 2 diabetes, and models for type 2 can be either obese or nonobese [98]. A comparison of the currently available genetic models can be found in Table 1.

#### Mouse Genetic Models of DR

There are five known genetic mouse models of DR: Ins2^Akita^, nonobese diabetic (NOD), db/db (Lepr^db^), Kimba, and Akimba. These models vary in mode of inheritance, disease etiology, pathology, and progression of disease. The *Ins2* ^*Akita*^ mouse is a model for type 1 diabetes that harbors a missense mutation in the *Insulin 2* gene. The missense mutation leads to a conformational change in the insulin protein, causing the protein to accumulate in pancreatic β cells, leading to β-cell death [99, 100]. Disease onset in this model is at 8 weeks, with an increase in retinal vascular permeability and reactive gliosis. Disease progression continues up to 8 months of age, with a reduction in axons and dendrites of RGCs by 12 weeks, an increase in acellular capillaries at 36 weeks, and an increase in leukocytes in the vascular wall, as a result of inflammation [101, 102]. Additionally, a decreased number of cholinergic and dopaminergic amacrine cells [103] is also observed leading to a reduction in the thickness of the IPL and INL [101, 104]. The *Ins2* ^*Akita*^ mouse is useful for studying the early progression of DR and the neuroprotective effects of treatments, as loss of RGCs can be detected in a short span of time [20].

A second commonly used model for type 1 diabetes is the NOD mouse, which exhibits an autoimmune response in which the CD4^+^ and CD8^+^ cells attack pancreatic β cells [21, 105, 106]. Diabetes in NOD mice has been well documented and, similar to humans, is a polygenic model with several loci associated with the disease phenotype [107–109]. Similar to what is observed in humans with type 1 diabetes, NOD mice suffer from infiltration of dendritic cells and macrophages in pancreatic islets leading to inflammation, hyperglycemia, and apoptosis of insulin-producing β cells [21, 22, 23•]. Disease onset begins when spontaneous hyperglycemia occurs in these mice by 12 weeks of age. NOD mice show apoptosis of pericytes, endothelial cells, and RGCs, as well as retinal capillary basement membrane thickening starting at 4 weeks [110]. Vasoconstriction and degeneration of major vessels with abnormal microvessels can be detected approximately 4 months after hyperglycemia [111]. Additionally, focal proliferation of vessels was also detected [112]. In contrast to the human disease, NOD mice exhibit a gender bias where by 30 weeks, 80% of females and 20% of males become diabetic [21]. Due to the variation of diabetes seen in females and males, constant monitoring of glucose levels is important for appropriate study design. Despite the gender bias, the pathophysiology of type 1 diabetes is very similar between the NOD mouse model and humans, making it a desirable model for DR research [113].

The db/db (*Lepr* ^db^) mice were developed to study type 2 diabetes. db/db mice harbor a mutation in the leptin receptor and develop hyperglycemia and obesity after 4–8 weeks [24, 25], and disease progression continues for 10 months. Homozygous animals exhibit chronic hyperglycemia, morbid obesity, atrophy of pancreatic β cells, and eventually become hypoinsulinemic [25, 114, 115]. After 6 weeks, a reduction in the number of RGCs and increased thickness in the central retina are observed [116]. By 18 weeks, reactive gliosis as well as pericyte loss is detected [117]. This model is used to study late stages of the disease as db/db mice present late reactive gliosis along with vessel leakages [118]. db/db mice have continued increases in blood sugar levels, severe depletion of pancreatic islets, and myocardial diseases, eventually leading to death at approximately 10 months of age [119, 120].

Another method to develop a more physiologically relevant mouse model for DR is breeding two mutant mouse strains. The Akimba mouse was generated by crossing two mouse strains. The Kimba mice, a nondiabetic model of proliferative retinopathy resulting from overexpression of *Vegf* driven by the rhodopsin promoter [121, 122], were crossed with the diabetic *Ins2* ^*Akita*^ mice to create the Akimba model. Kimba mice show reduction in the INL and ONL by postnatal day 7 (P7) [123]. By P28, microvascular abnormalities and capillary dropout are observed and continue until 9 weeks of age, at which time pericyte loss is detected [123, 124]. The Akimba mice are hyperglycemic and appear to have additive effects from both parental strains [125, 126]. Akimba mice are characterized by pericyte and vessel loss and retinal neovascularization with diffuse vascular leakage that is observed in late-stage DR [125, 127]. Additionally, the Akimba mouse exhibits leaky capillaries, tortuous vessels, and microaneurysm by 8 weeks [125]. Enhanced photoreceptor loss, reduction of retina thickness, increased persistence of edema, and retinal detachment are observed as the animal ages and disease progresses [125].

#### Rat Genetic Models of DR

There are six genetic rat models of DR: Zucker diabetic fatty (ZDF), Otsuka Long-Evans Tokushima fatty (OLETF), biobreeding (BB), WBN/Kob, spontaneously diabetic Torii (SDT), and Goto-Kakizaki (GK). The ZDF, OLETF, and BB are monogenic models of DR with independent mutations that perturb different nodes of the DR disease pathway (Fig. 1). The SDT, WBN/Kob, and GK models, in contrast, are polygenic models. These models demonstrate the genetic complexity of DR. Quantitative trait locus (QTL) analysis identifying novel DR loci may provide insight into the multiple genes and networks that are impacted and lead to DR pathogenesis in humans.

There are three monogenic rat models of DR. The ZDF rat is a monogenic model for severe spontaneous type 2 diabetes. These animals have a missense mutation in the leptin receptor gene, *Lepr*, that results in insulin tolerance along with excessive body weight gain [128, 129]. ZDF rats are characterized by hyperglycemia at 6 weeks that continues throughout their lives. As a result, thickening of the capillary basement membrane can be detected along with increased capillary cell nuclear density 5 months after hyperglycemia [130].

The OLETF rat is a monogenic DR model that was created by the selective breeding of Long-Evans rats that is characterized by obesity, hyperglycemia, and glycosuria [26]. OLETF is a model for spontaneous type 2 diabetes and obesity and harbors a mutation in the initiation codon of the G-protein-coupled receptor GPR10 that leads to obesity [131]. Disease onset is characterized by increased blood glucose that is observed by 5–6 months [132]. Six weeks following the onset of hyperglycemia, microvessel-related symptoms are observed, including leukocyte entrapment in retinal microcirculation [26]. At 3 months posthyperglycemia, a reduction in the number of pericytes is detected and damage of endothelial cells is observed [133]. In addition, increased thickness of the capillary basement membrane, microaneurysms, capillary formation in loops, and tortuosity are also detected [26, 134]. This model is not commonly used to study DR as it lacks acellular capillaries and has a late onset of diabetes.

The BB monogenic model is widely used to study type 1 diabetes. This model presents with retinal lesions, pericyte loss, capillary degeneration, and microaneuryms by 8–11 months, as well as apoptosis of pancreatic β cells due to an autoimmune response [135, 136]. The BB model harbors a frameshift mutation in the immune-associated nucleotide-binding protein gene *Ian4*, also known as *Ian5*, *Iddm1*, and *Gimap5*. The mutation generates the lymphopenia phenotype associated with diabetes [137-139]. Alterations in lymphopenia are associated with both type 1 and type 2 diabetes [140, 141]. Lymphophenia is also commonly observed in cancer and autoimmune disease [142–144].

The WBN/Kob rat model presents acellular capillaries and is a spontaneous model of type 2 diabetes whose causative gene remains unknown [20, 145]. This model is characterized by intraretinal angiopathy with new vessel formation and hyalinization of intraretinal vessels, making it an ideal model for understanding the progression of DR [146]. To determine the gene or genes involved in generating the described phenotype in this model, QTL analysis identified two significant regions in chromosome 7 and X hinting that several genes might be involved [147]. Later studies were able to narrow down the search to chromosome 7, a region designated as Pdwk1 (pancreatitis and diabetes mellitus in WBN/Kob locus 1) that harbors 14 genes [148].

There are two rat models of nonobese type 2 diabetes: the SDT and the GK rats. The SDT male rats develop glycosuria at approximately 20 weeks compared to females at 45 weeks [149, 150]. Similarly diabetes develops at different rates: by 40 weeks, 100% of males have diabetes, compared to 33% of females at 65 weeks when the study ended [150]. SDT rats are characterized by retinal dysfunction, which includes retinal detachment with fibrous proliferation, absence of retinal ischemia in the presence of neovascularization, leukostasis, increased number of apoptotic cells in the GCL and INL, vascular lesions, and pericyte loss [151–154]. A distinct feature of this model is the appearance of large retinal folds with extensive leakage around the optic disc, similar to retinal detachment observed in humans [151–154]. The SDT rat is the rat model that most closely resembles the pathophysiology seen in humans; however, the absence of microaneurysm makes them a more suitable model for studying NPDR. The characteristic phenotype associated with SDT rats has been linked to three QTL assigned as Gisdt1, Gisdt2, and Gisdt 3 for glucose intolerance found in chromosomes 1, 2, and X, respectively [155].

GK rats, in contrast to SDT rats, develop hyperglycemia earlier at 4–6 weeks and have an increased number of retinal endothelial cells compared to the number of pericytes present [156, 157]. GK rats are characterized by having a reduced retinal blood flow with no changes in the diameter of veins and arteries in the early stages of diabetes, making it an excellent model to study microcirculatory changes in the retina [20, 158]. Increased BRB permeability is observed at 3 months followed by increased endothelial/perycite ratio at 7 months [158, 159]. GK rats were generated by multiple inbreeding crosses of the glucose-intolerant Wistar rats. Consequently, the exact genetic background as well as the causative genes remains unknown [160]. Whole genome sequencing and QTL analysis revealed 192 potential genes [161].

#### Zebrafish Genetic Model of DR

Zebrafish are valuable genetic models for human diseases as genetic manipulation is easily performed and they often recapitulate human retinal vascular disease accurately. The *Vhl* zebrafish have a mutation in the von Hippel-Lindau tumor suppressor gene and are characterized by increased blood vessel formation, along with upregulation of the hypoxia-inducible factor, which triggers expression of *Vegf* [162]. This model is characterized by an increased number of hyaloid vasculature with concomitant vascular leakage, macular edema, retinal detachment, and severe neovascularization [151].

## Conclusions and Future Directions

Animal models play a crucial role in understanding the etiology and pathophysiology of DR and in the development of viable therapeutics to prevent and attenuate disease. DR is a complex disease that involves multiple genetic and environmental inputs and, therefore, a challenging disease to model. Most models focus on a genetic or environmental insult to one of the major DR phenotypes. Combining genetic and/or genetic and induced models may provide more accurate DR models. An example is the generation of the Akimba mouse that results from breeding Kimba mice, which overexpress *Vegf*, with the Akita mice, which have spontaneous type 1 diabetes designed to generate a model that has the key characteristic of the early and late phases of the disease and as many traits of the phenotype as possible. As of today, the available models, both induced and genetic, are mostly characteristic of NPDR, such as microaneurysm and retinal degeneration, and key characteristics of PDR, such as neovascularization, are less likely to be seen in the models. Similarly, the majority of models imitate the aspects of early DR, and only few of the high-order animals recapitulate the retinopathies of the later stages of the disease.

**Fig. 2:**
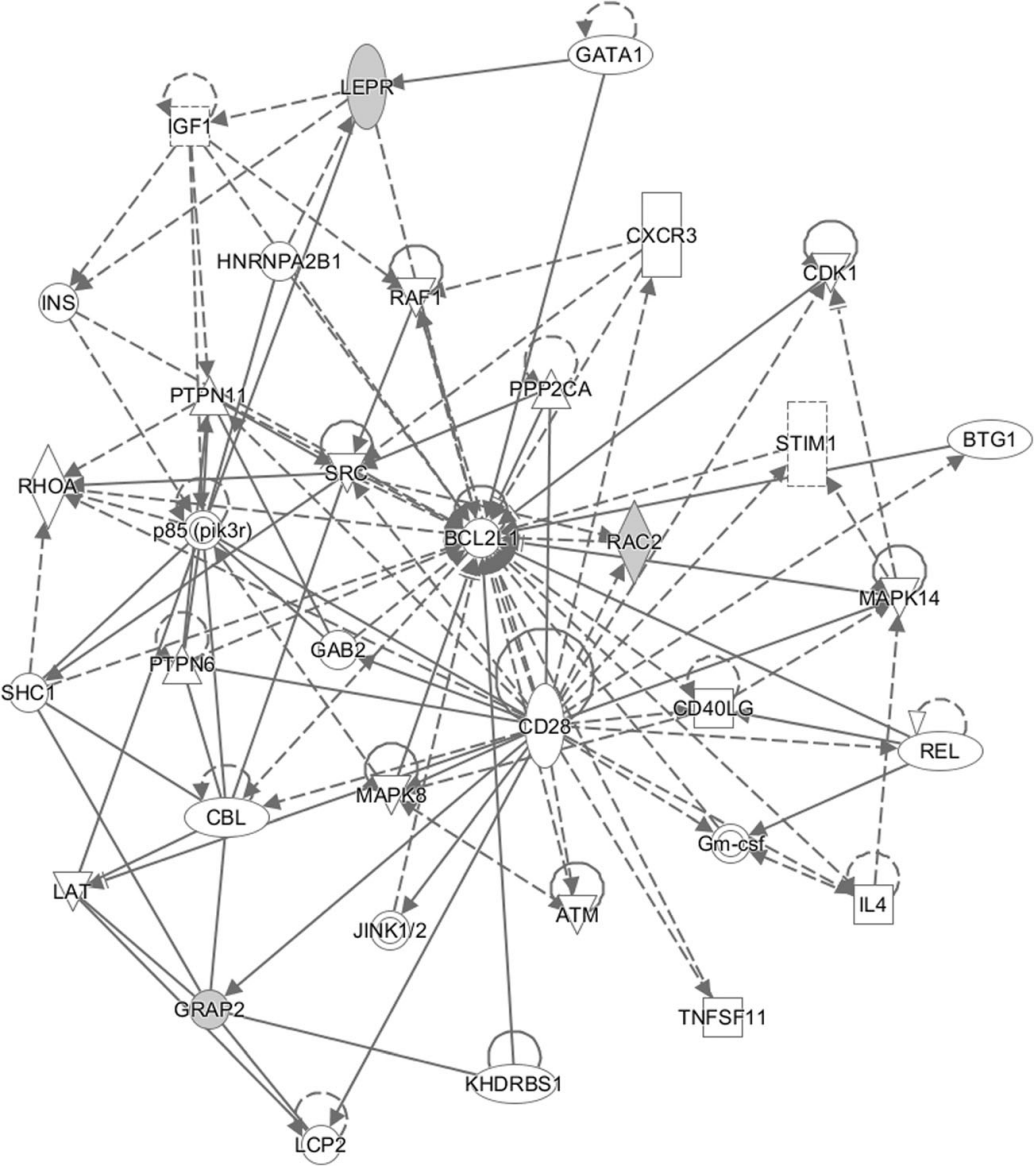
Hypothetical diabetic retinopathy gene network. Ingenuity pathway analysis of genes reviewed in this article yielded one major gene network that contains genes that fall under the following biological classifications: for cellular development, growth, proliferation, and lymphoid development and structure

The majority of the available models better recapitulate the early stages of the diseases, limiting the availability of models to evaluate comprehensive therapies for DR. Treatments are generally restricted to targeting the early progression of the disease due to the available models. Additionally, animal models of retinal neovascularization, without hyperglycemia, have been developed. These models may provide valuable tools to understand pathogenesis and develop appropriate treatment options for late-stage DR disease. It is critical to properly understand the pathophysiology and limitations of each available model to determine the best model for a study. Based on current research, we generated a hypothetical DR gene network (Fig. 2) [163]. Data were analyzed through the use of IPA (QIAGEN Inc., https://www.qiagenbioinformatics.com/products/ingenuity-pathway-analysis). The review and hypothetical network will provide bases for improved therapeutic study design to develop viable treatments for this complex and common disease.

## Abbreviations

BB: Biobreeding rat: widely used rat model of diabetes
BRB: Blood retinal barrier: wall of tightly joined cells that prevent the movement of substances from blood into the retina
DME: Diabetic macular edema: leaking macular capillaries that lead to loss of vision associated with diabetes
DR: Diabetic retinopathy: damage to the retina leading to severe vision loss caused by diabetes
GCL: Ganglion cell layer: inner most retina layer comprised of ganglion cells and displaced amacrine cells
GLUT2: Glucose transporter 2: carrier protein that transports glucose in the liver and blood
GK: Goto-Kakizaki rat: rat model with hyperglycemia
GPR10: G-protein-coupled receptor: protein that binds to prolactin-releasing peptide
INL: Inner nuclear layer: nuclear layer in the inner retina comprised of bipolar, horizontal, and amacrine cells
IPL: Inner plexiform layer: layer of neuronal synapses connecting the bipolar and amacrine cells to ganglion cells
MESA: Multi-Ethnic Study of Atherosclerosis: medical research study involving 6000 men and women from the six continents
NOD: Nonobese diabetic mouse: mouse model of diabetes that is characterized by the absence of weight gain
NPDR: Nonproliferative diabetic retinopathy: first stage of diabetic retinopathy characterized for the absence of symptoms
ONL: Outer nuclear layer: layer from the retina involve in the detection of the light
OIR: Oxygen-induced retinopathy: retinopathy caused by the exposure to oxygen concentrations in a chamber
OLETF: Otsuka Long-Evans Tokushima fatty rat: rat model characterized by obesity and hyperglycemia
PSL: Photoreceptor segment layer: retina layer composed of rod and cones.
P: Postanatal: related to the time period after birth
PDR: Proliferative diabetic retinopathy: advance stage of diabetic retinopathy characterized for the increase of new blood vessels that eventually leak
QTL: Quantitative trait locus: segment of DNA that correlates to a variation in phenotype
RGC: Retinal ganglion cell: neuron present in the inner surface of the retina
SDT: Spontaneously diabetic Torii rat: rat models of nonobese type 2 diabetes
STZ: Streptozotocin: chemical that is toxic to the insulin producing β cells in the pancreas
VEGF: Vascular endothelial growth factor: signal protein involved in angiogenesis
ZDF: Zucker diabetic fatty rat: rat model with spontaneous diabetes
